# Sphingolipids mediate lipotoxicity in muscular dystrophies

**DOI:** 10.1093/lifemedi/lnac015

**Published:** 2022-06-28

**Authors:** Yuefan Wang, Ng Shyh-Chang

**Affiliations:** State Key Laboratory of Stem Cell and Reproductive Biology, Institute of Zoology, Chinese Academy of Sciences, Beijing 100101, China; Institute for Stem Cell and Regeneration, Chinese Academy of Sciences, Beijing 100101, China; University of Chinese Academy of Sciences, Beijing 100049, China; State Key Laboratory of Stem Cell and Reproductive Biology, Institute of Zoology, Chinese Academy of Sciences, Beijing 100101, China; Institute for Stem Cell and Regeneration, Chinese Academy of Sciences, Beijing 100101, China; University of Chinese Academy of Sciences, Beijing 100049, China

Muscular dystrophies are a genetically diverse group of muscular diseases that share similar clinical symptoms of progressive weakness and death of skeletal muscle cells during development. Over 30 different disorders are classified as muscular dystrophies, including Becker muscular dystrophy (BMD), Duchenne muscular dystrophy (DMD), Emery–Dreifuss muscular dystrophy (EDMD), limb–girdle muscular dystrophy, and myotonic muscular dystrophy—all of which are themselves diverse in the age of onset, the speed of pathogenesis, the degree of weakness, the exact muscle groups that are affected, and the gene mutations that are involved [1]. Nevertheless, most if not all muscular dystrophies are caused by inherited or spontaneous genetic mutations that implicate aberrant muscle protein expression, e.g. dystrophin for BMD and DMD, and dysferlin for distal muscular dystrophy.

Thus, it stands to reason to attempt to treat all muscular dystrophies with gene and cell therapies that correct the particular genetic mutation latent within the patient. Efforts in this area include viral transduction of microdystrophin, delivery of CRISPR gene editors or antisense RNAs that suppress pathogenic gene expression, or myoblast transfer therapy, i.e. autologous injections of gene-corrected myogenic stem cells to mediate normal muscle regeneration. However, these gene and cell therapeutic approaches are mired in two quandaries. Firstly, the huge diversity of mutations within each type of muscular dystrophy complicates the applicability of off-the-shelf therapies. Secondly, the small numbers of patients available for each genotype of muscular dystrophy complicates clinical trial design. While these problems are being solved, other pharmacological solutions have also been developed to ameliorate muscular dystrophies, including anabolic steroids, anticonvulsants, calcium channel blockers, and immunosuppressive corticosteroids. Such non-genetic drug treatments could greatly help patients where gene therapies are not suitable or available yet, and could still complement gene therapies in the future.

**Figure 1.**
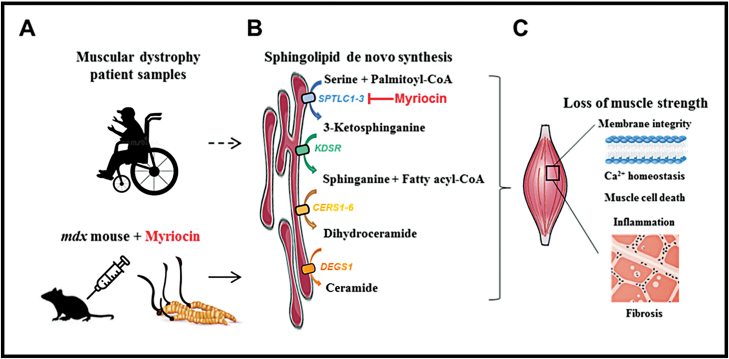
Schematic of how the sphingolipid biosynthesis pathway was discovered and verified to be important in muscular dystrophies. (A) Laurila et al. (2022) used muscle biopsies from patients with various types of muscular dystrophy for transcriptome profiling and revealed that they show common aberrations in sphingolipid biosynthesis enzyme expression. The *mdx* mouse model of Duchenne muscular dystrophy reflected this correlation as well in many cell types. (B) Treatment with myriocin, an inhibitor of serine palmitoyltransferase (SPTLC1-3) derived from *Cordyceps* and thermophilic fungi, effectively reduced many of the downstream metabolites produced by 3-ketodihydrosphingosine reductase (KDSR), ceramide synthases (CERS1-6), and dihydroceramide desaturase 1 (DEGS1) in sphingolipid biosynthesis. (C) Many of the complex phenotypes reflected in the *mdx* mouse model of Duchenne muscular dystrophy, including loss of membrane integrity, calcium (Ca^2+^) homeostasis dysregulation, muscle cell death, inflammation mediated by M1 macrophages and fibrosis mediated by fibro-adipogenic precursors, were reversed more effectively by myriocin treatment than an immunosuppressive glucocorticoid.

While genetically diverse, all muscular dystrophies commonly involve myofiber degeneration, aberrant calcium homeostasis, cell death, inflammation and fibrosis [2]. Interestingly, sphingolipids are lipid messengers which regulate calcium signaling, cell death, inflammation, and fibrosis as well [3]. Ceramides and sphingomyelins are products of sphingolipid metabolism, synthesized from the acylation of sphinganine, which is in turn a condensation product of serine with palmitate through the sphingolipid de novo synthesis pathway [4]; (Fig. 1). Ceramides can regulate the balance between autophagy, senescence, and apoptosis. They can also be de-acylated and phosphorylated to form sphingosine-1-phosphate, which can bind to G-protein coupled receptors (GPCRs) to regulate mitosis, calcium signaling, and development. In a set of surprising observations, Laurila et al. recently found a correlation between sphingolipid biosynthesis and muscular dystrophies [5]. Enzymes in the sphingolipid biosynthesis pathway were upregulated in the skeletal muscle biopsies of patients with different types of muscular dystrophy. Furthermore, based on liquid chromatography-mass spectrometry (LC-MS), DMD patient myoblasts showed an accumulation of sphinganine, dihydroceramides and ceramides—the intermediates and products of the sphingolipid de novo synthesis pathway. Moreover, the *mdx* mouse model of DMD also showed higher levels of sphinganine, dihydroceramides and long-chain ceramides in their quadriceps muscles, macrophages and plasma, compared to wild-type mice. These results suggested a conserved upregulation of sphingolipid biosynthesis across multiple cell-types implicated in muscular dystrophies (Fig. 1).

The rate-limiting enzyme of sphingolipid metabolism is serine palmitoyltransferase (SPT), where two subunits of the enzyme SPTLC1 and SPTLC2 were upregulated in *mdx* mouse muscles as well. Pharmacological inhibition of sphingolipid biosynthesis can be achieved via myriocin, an antibiotic inhibitor of SPT [6]. Myriocin is also known as thermozymocidin, a non-proteinogenic amino acid metabolite derived from thermophilic fungi, as well as the entomopathogenic fungi (*Cordyceps* genus) that has long been used in traditional Chinese medicine. Scientifically, it has been shown to possess highly potent immunosuppressant properties. It is a part of the molecular armamentarium through which entomopathogenic fungi attack living insects, presumably by suppressing their immune system as well. In the *mdx* mouse model of DMD, myriocin inhibition of SPT reduced the high levels of sphinganine, dihydroceramides and long-chain ceramides, thereby improving calcium homeostasis, reducing intramuscular inflammation and inflammation-induced cycles of muscle regeneration. Notably, SPT inhibition shifted the balance from pro-inflammatory M1 macrophages towards anti-inflammatory M2 macrophages, and suppressed fibrosis mediated by PDGFRα+ fibro-adipogenic precursors (FAPs) in the tibialis anterior muscles, diaphragm muscles and cardiac muscles, thereby preventing the loss of muscle strength, respiratory failure and cardiac failure in DMD. At low concentrations (which avoided the hepatotoxicity occasionally observed in rodents), myriocin still rescued these *mdx* mouse phenotypes more effectively than a corticosteroid drug (Fig. 1). This groundbreaking study demonstrated that inhibition of sphingolipid biosynthesis can target multiple common mechanisms in muscular dystrophies simultaneously, indicating that this metabolic pathway could represent a series of druggable targets for the development of novel treatments against muscular dystrophies.

As these findings begin to spark a new wave of exciting developments in therapies against muscular dystrophies, it is worth noting that the fatty acid-derived ceramides have long been suspected as a driver of lipotoxicity in the metabolic syndrome (MetS) during human aging. Dysfunction in lipid metabolism and especially hyperlipidemia are key risk factors that lead to a variety of MetS diseases associated with insulin resistance (IR), such as myosteatosis, sarcopenic obesity, cachexia, non-alcoholic steatohepatitis, cardiomyopathy, and type 2 diabetes [7]. For instance, a high flux of fatty acids into ceramides could suppress Akt phosphorylation and cause IR [8]. Conversely, drug inhibitors for ceramide and sphingolipid biosynthesis have shown promising results in mouse models of IR *in vivo* [8]. In contrast, sphingosine-1-phosphate and its related metabolites can regulate cell proliferation and cell death in many tissues. How do these sphingolipids cross-talk with each other in multiple cell-types across the various tissues? Does this metabolic pathway represent a common thread that underlies stem cell senescence during both MetS and muscular dystrophies? This would be an intriguing possibility, given that there is emerging evidence that muscular dystrophy patients also exhibit IR and myosteatosis [9]. Could the Metabaging Cycle, i.e. local myoblast and myofiber inflammation leading to local IR and dyslipidemia, ultimately leading to a lipotoxic vicious cycle that gradually impairs systemic health during aging [10], occur in both MetS and muscular dystrophy patients via sphingolipid-mediated toxicity? More work will be needed to unravel these common threads weaving the complex tapestry of metabolic dysfunction in muscle disorders.
